# Associations of Resilience With Bonding Status After Preterm Birth

**DOI:** 10.7759/cureus.95791

**Published:** 2025-10-31

**Authors:** Sayuri Harada, Kumie Kataoka, Nobuyuki Miyatake, Mikiya Nakatsuka

**Affiliations:** 1 Department of Maternity Health Nursing/Midwifery, Faculty of Medicine, Kagawa University, Kagawa, JPN; 2 Department of Nursing, Faculty of Health and Welfare, Kawasaki University of Medical Welfare, Okayama, JPN; 3 Department of Hygiene, Faculty of Medicine, Kagawa University, Kagawa, JPN; 4 Department of Nursing, Graduate School of Health Sciences, Okayama University, Okayama, JPN

**Keywords:** bonding, infant, mothers, preterm birth, resilience

## Abstract

Preterm birth can affect maternal-infant bonding; however, the factors contributing to bonding after preterm delivery remain poorly understood. The purpose of this study was to determine the impact of maternal resilience and the ability of mothers to adjust to the difficulties after preterm birth on the bonding process. To this end, the impact of resilience on bonding and the ability of mothers to adjust to difficulties after preterm delivery was evaluated across four maternity facilities in Okayama Prefecture, Japan, between July 2015 and July 2017. A regression analysis revealed that resilience was the most important maternal bonding factor in the preterm group (β = 0.50, *p* = 0.00), followed by social support from the husband (β = 0.31, *p* = 0.03). The results suggest that strengthening the resilience of mothers who experienced preterm birth could strengthen bonding.

## Introduction

Premature birth is the most frequently encountered event in the Neonatal Intensive Care Units (NICUs). Japan’s preterm birth rate is 5.8%, lower than the 8-12% rate in Western countries. Furthermore, the mortality rate for preterm births at less than 32 weeks’ gestation and birth weight under 1500 g is 5% in Japan, lower than in Western countries (12% in the US, 8% in Canada). Japan's perinatal mortality rate is among the lowest in the world [1]. Premature infants, due to their immaturity, face complications such as chronic lung disease, retinopathy of prematurity, and necrotizing enterocolitis, as well as long-term neurodevelopmental issues [1], often requiring long-term follow-up.

Maternal complications (including preeclampsia, the primary cause of iatrogenic preterm birth) increase in incidence with age. Consequently, the number of women becoming pregnant at an advanced age has risen in recent years, leading to a global increase in the proportion of older pregnant women, including in Japan [2]. Like preterm infants, these mothers often require ongoing health management.

Preterm birth is an unexpected event that requires medical intervention for the newborn, typically resulting in a separation between the mother and the child. When giving birth prematurely, the role of the mother develops during a period of uncertainty regarding the child's prognosis, requiring adaptation to the unique needs and characteristics of the preterm child. Emotional trauma during preterm delivery affects the ability of a mother to assume this role and to develop her maternal identity [3].

The psychological effects of preterm delivery on mothers include a sense of loss of a “normal” birth, acute emotional responses, guilt feelings [4], and shock of rejection [5]. Thus, preterm delivery contributes to postpartum depression. Furthermore, postpartum depression is reported to be associated with neonatal abuse [6,7]; therefore, preterm birth is a risk factor for abused mothers. In addition, preterm infants are more vulnerable to abuse due to their slower physical and cognitive development, difficulties in feeding, and challenges in interpreting and responding to their behavioral cues [8].

Bonding refers to the affection between parents and their children that is fostered during pregnancy and the raising of children [9]; this concept was proposed by Klaus and Kennel and subsequently evaluated [3,10-13]. Perinatal factors that may adversely affect maternal bonding include severe postpartum hemorrhage and premature placental separation. These complications are particularly important when the infant is admitted to the NICU, and the mother and child are separated [14]. Preterm deliveries often lead to emergency deliveries or cesarean sections due to the fragile state of the fetus and sudden deterioration of the condition of the child, and there is evidence that such deliveries may impair maternal bonding [15,16]. In addition, an association between maternal bonding and postpartum depression has been reported [7]. Thus, preventing bonding disorders and emotional bonding between mothers and children is crucial after preterm delivery.

Resilience is the process of successfully adapting to adversity when faced with trauma, threats, or significant sources of stress [17]. Resilience can be strengthened not only by individual characteristics but also by effective support from one’s surroundings [18]. Resilience related to parenting by mothers has been reported to be lower for single parents and young mothers raising children [19], whereas resilience in mothers of children with disabilities tends to increase with adequate support [20,21]. However, studies of the resilience of mothers who give birth prematurely are lacking. This study evaluated the relationships between various factors, including resilience, social support, postpartum depression, and bonding among preterm mothers.

## Materials and methods

Research design

A cross-sectional quantitative descriptive study was conducted using a self-administered, unmarked questionnaire.

Data collection

The study was conducted between July 2015 and July 2017. It involved four obstetric facilities in Okayama, Japan. Mothers who gave birth prematurely at 28 weeks or less than 37 weeks of gestation (preterm group) were included. To clarify differences in attachment and its associated factors between preterm and full-term infants, a study was conducted comparing these two groups, which exhibit significant differences in the process leading to delivery and infant condition, while also including mothers who gave birth to full-term infants. Mothers who gave birth full-term (full-term group) were included as a comparison. Mothers whose babies had congenital defects, morphological abnormalities, and serious health conditions(e.g., facial, cardiac, or gastrointestinal malformations; abdominal wall hernias; abnormalities in brain shape or structure), and who could not communicate adequately in Japanese were excluded. If the child is a multiple birth, information on the first child was obtained.

In total,166 mothers (66 in the preterm infant group, 100 in the term infant group) participated in the study. For the term infant group, midwives in the obstetrics wards at each of the four facilities provided written and verbal explanations of the study outline. Participants who gave consent completed an anonymous self-administered questionnaire during their hospital stay (1 to 5 days postpartum) and deposited it in a collection box within the ward before discharge. In the preterm infant group, the administrator contacted the researcher, who then provided written and verbal explanations, taking into account the mother's mental state. Mothers who consented completed the anonymous self-administered questionnaire during hospitalization (3 to 8 days postpartum) and either deposited it in a collection box on the ward or mailed it.

Measurements

Background of Participants

Demographic characteristics included maternal and partner age, pre-pregnancy status, mode of delivery, gestational age at delivery, infant birth weight, and singleton versus multiple pregnancy.

Maternal Attachment Inventory (Japanese Version)

The Japanese version of the Maternal Attachment Inventory (MAI-J), a questionnaire, was used to measure mother-to-infant attachment to evaluate bonding [22]. The MAI-J is a 26-item, 4-point Likert rating scale with scores ranging from “rarely” (1 point) to “almost always” (4 points) and total scores ranging from 26 to 104, with higher scores indicating greater maternal affection toward the infant. The higher the score, the more affection the mother exhibited for the infant. Cronbach's alpha coefficient in this study was 0.92. This scale was used in the research with the authors’ consent.

Bidimensional Resilience Scale (BRS)

Resilience was measured using the BRS [23]. The resilience score was composed of innate resilience factors (iR) (9-45 points) and acquired resilience factors (aR) (12-60 points), with higher scores indicating greater resilience to recovery from psychological injury. Cronbach's alpha coefficient was 0.90 for the overall scale, 0.83 for the innate resilience factor subscale, and 0.72 for the acquired resilience factor subscale. This measure was selected for the study based on the belief that resilience is a trait that can be cultivated and strengthened, and we aimed to actively support that development. This scale was used in the research with the authors’ consent.

Social Support

The social support scale was developed by the author and encompasses inputs from husbands, parents (biological parents and parents-in-law), friends, and mothers with firsthand experience of preterm birth. Participants rated various social support factors using a 4-point Likert scale. The scores were as follows: “not often” (1 point), “sometimes” (2 or 3 points), and “always” (4 points). The reliability of the scale, measured by Cronbach's alpha coefficient, was 0.821 for the entire scale and ranged from 0.626 to 0.831 for each factor.

Edinburgh Postnatal Depression Scale (EPDS)

The Japanese version of the Edinburgh Postpartum Depression Questionnaire, a self-administered questionnaire consisting of 10 items and 4 tests, was used to assess postpartum depression. A score of 0-3 points was assigned to each questionnaire response, with a minimum score of 0 and a maximum score of 30. The classification score in the Japanese version is 8/9; based on Japanese data, a score of 9 or higher indicates a high risk of postpartum depression [24]. Cronbach’s alpha coefficient was 0.78. This scale was used in the research with the authors’ consent.

Statistical analyses

Comparisons of preterm and full-term birth groups were evaluated using χ² tests, t-tests, and Pearson's correlation analyses. To clarify the influence of each variable, a multiple regression analysis was conducted, using bonding as a dependent variable. Statistical analyses were performed using IBM SPSS Statistics for Windows v.28 (IBM Corp., Armonk, NY, USA).

Ethical consideration

The purpose of the research, the fact that cooperation was voluntary, the fact that non-co-operation would not have negative consequences (e.g. involving the use or content of medical services), the protection of personal information, and of anonymity in the disclosure of results were all explained to the participants in writing and orally, and each of them gave their consent to participate and return the filled-up survey form. This study was approved by the Ethics Review Committee of the Graduate School of Health Sciences at Okayama University (No. D14-04).

## Results

Characteristics of the participants

Among the 166 eligible mothers, 112 (67.5%) replied to the questionnaire (Table 1). Of these, 104 mothers, including 40 in the preterm group and 64 in the full-term group, completed the survey without missing data for key variables and were included in the analysis (valid response rate: 62.7%).

**Table 1:** Characteristics of the Participants No mark: χ^²^ test, a: t-test, V: Cramer’s V Values are expressed as mean(SD), unless otherwise specified.

	Preterm (n=40)	Full-term (n=64)	p-value and others
Mother’s Age: years old	34.8 (6.4)	30.7 (6.4)	0.00^a^
Father’s age: years old	36.9 (9.3)	31.4 (7.0)	0.00^a^
Parity(%)			
Primiparae	27 (67.5)	54 (84.4)	0.45
Multiparae	13 (32.5)	10 (15.6)	χ^2^(1)=6.11, V=0.24
The way of conceiving			
Naturally (%)	30 (78.9)	42 (72.6)	0.64
Infertility (%)	8 (21.1)	17 (27.4)	χ^2^(1)=0.04, V=0.02
The way of birth			
Vaginal birth	20 (50.0)	50 (78.1)	0.00
Cesarean planned	5 (12.5)	11 (17.2)	χ^2^(2)=19.09, V=0.43
Cesarean emergency	15 (37.5)	3 (4.7)	
Gestational age: weeks	33.0 (2.6)	39.5 (1.3)	0.00^a^
Birth weight:g	1869 (453.8)	3005 (288.7)	0.00^a^
Singleton	32 (80)	64 (100)	0.01^a^
Multiple	8 (20)	0	

The age of mothers (mean ± S.D.) in the preterm group was 34.8 ± 6.4 years, and that of fathers was 36.9 ± 9.3 years. Both these estimates were significantly higher than those of the full-term mothers and fathers. There were eight multiples in the preterm group and none in the full-term group. The rate of emergency cesarean section was 37.5% in the preterm group, significantly higher than in the full-term group. The duration of hospitalization during pregnancy was significantly longer in the preterm group compared to the full-term group.

Comparison of variables between the preterm group and the full-term group

No significant difference in bonding was observed between the preterm and term groups. The preterm group had significantly higher scores on the Brief Resilience Scale, which measures general resilience, and on the acquired resilience (aR) subscale. The EPDS score was significantly higher in the preterm group compared to the term group (Table 2).

**Table 2:** Comparison of variables t test EPDS: Edinburgh Postnatal Depression Scale; BRS: Bidimensional Resilience Scale; MAI-J: Maternal Attachment Inventory Japanese Version

	Preterm (n=40)	Full-term (n=64)	ｐ-value
Bonding (MAI-J)	78.3±10.3	77.3±10.4	0.638
Resilience (BRS) total BRS	77.1±10.4	75.5±9.9	0.000
Acquired resilience	35.3±4.2	33.8±9.7	0.000
Innate resilience	44.3±6.4	43.3±7.2	0.074
Social support of husband	3.2±1.1	3.1±1.0	0.532
Social support of parents	3.2±1.0	3.2±1.0	0.175
Social support from friends	2.5±1.1	2.4±0.9	0.712
Social support experienced preterm birth	1.9±1.1	2.1±1.1	0.309
Postpartum depression(EPDS)	7.4±5.4	5.0±4.6	0.001

Correlation between bonding and each variable

In the preterm group, maternal bonding showed a weak negative correlation with both mother's age (r = -0.292, p = 0.047) and number of children (r = -0.129, p = 0.048). Bonding was positively associated with all resilience dimensions-total, acquired, and innate-with moderate correlation coefficients ranging from r = 0.517 to 0.578 (p = 0.000-0.001). Additionally, a weak positive correlation was observed between bonding and perceived social support from the husband (r = 0.355, p = 0.031) (Table 3).

**Table 3:** Correlation between bonding and each variable r: correlation coefficient; MAI-J: Maternal Attachment Inventory Japanese Version; BRS: Bidimensional Resilience Scale; EPDS: Edinburgh Postnatal Depression Scale

	Bonding (MAI-J)			
	Preterm (n=40)		Full-term (n=64)	
	r	p	r	p
Mother’s age	-0.292	0.047	-0.194	0.088
Number of children	-0.129	0.048	－ －	
Gestational age	-0.171	0.291	0.056	0.659
Birth weight	-0.211	0.192	-0.207	0.101
Resilience (total BRS)	0.587	0.000	0.792	0.087
Acquired resilience	0.578	0.000	0.675	0.321
Innate resilience	0.517	0.001	0.775	0.054
Social support (Husband)	0.355	0.031	0.038	0.771
Social support (Parents)	0.038	0.822	0.355	0.031
Social support (Friends)	0.013	0.940	0.125	0.955
Social support (experienced preterm birth)	0.086	0.614	0.167	0.197
Postpartum depression (EPDS)	-0.056	0.741	-0.166	0.189

Factors Affecting Bonding

Bonding was set as the dependent variable, and variables showing significant correlation with bonding were selected as independent variables in all the multiple regression analysis in the Preterm group and Full-term groups. Furthermore, although no correlation was observed between EPDS and Bonding, EPDS scores were significantly higher than those of the term group; therefore, EPDS was included as an independent variable in this analysis. To avoid multiple covariates, the variables with the highest correlation coefficients within the same scale have been selected (Table 4).

**Table 4:** Hierarchical multiple regression of factors associated with Bonding (MAI-J) in the preterm group VIF: variance inflation factor; DW: Durbin-Watson; Bonding(MAI-J): Maternal Attachment Inventory Japanese Version; Resilience acquired: a subscale of the Bidimensional Resilience Scale

	Model Ⅰ			Model Ⅱ			Model Ⅲ			Model Ⅳ			Model Ⅴ				n=40
	β	p	95%CI	β	p	95%CI	β	p	95%CI	β	p	95%CI	β	p	95%CI		VIF
Mother’s age	-0.30	0.07	[0.99,0.03]	-0.32	0.05	[-1.05,0.01]	-0.24	0.14	[-0.92,0.13]	-0.09	0.55	[-0.66,034]	-0.11	0.45	[-0.66,0.30]		1.25
Number of infants	0.13	0.40	[-4.69,11.33]	0.10	0.53	[-5.75,10.94]	0.08	0.60	[-5.94,10.13]	0.10	0.47	[-4.48,9.55]	0.12	0.39	[-3.93,9.93]		1.09
Postpartum depression(EPDS)				-0.12	0.49	[-0.86,0.42]	-0.08	0.61	[-0.78,0.46]	0.07	0.62	[-0.43,0.71]	0.07	0.66	[-0.44,0.68]		1.26
Social support(husband) (A)							0.32	0.05	[-0.02,6.08]	0.31	0.03	[0.29,5.61]	0.28	0.05	[0.03,5.32]		1.10
Resilience: acquired(B)										0.50	0.00	[0.49,1.88]	0.49	0.00	[0.49,1.86]		1.18
A×B													-0.19	0.15	[-0.29,0.04]		1.03
Adjusted R^2^	0.05(F=2.116,p=0.135)			0.04(F1.556=,p=0.217)			0.12 (F=2.283,p=0.08)			0.33(F=4.823,p=0.002)			0.30(F=4.522,p=0.002)			DW=2.101	
ΔR^2^	0.10(F=2.116,p=0.135)			0.01(F0.492=,p=0.488)			0.09(F=4.068,p=0..51)			0.21(F=12.091,p=0.001)			0.04(F=2.179,p=0.149)				

In the preterm group, hierarchical multiple regression analysis revealed that model VI - incorporating acquired resilience (β = 0.50, p = 0.00) and social support from the husband (β = 0.31, p = 0.03)-accounted for the largest proportion of variance in bonding outcomes (Adjusted R² = 0.33, p = 0.002). To address the potential impact of the interaction between resilience (aR) and social support (husband), centering of both variables was performed, and the interaction term "Social support (husband) × Resilience (acquired)” was established. However, the interaction term input into model V showed no significant association with the dependent variable, and the Durbin-Watson statistic was 2.17, indicating no correlation between the independent variables. The full-term group did provide an important model for this analysis.

## Discussion

Associations of resilience with bonding status after preterm birth

During the first week postpartum, aR had the greatest impact on maternal bond formation after preterm birth, followed by spousal support. The resilience scale used in this study measures two aspects: innate and acquired resilience, with aR representing acquired resilience. This may reflect the needs of mothers who experienced preterm birth to adapt to overcome critical situations such as sudden delivery, numerous unexpected medical interventions, and mother-infant separation. This study revealed that acquired resilience most strongly influenced attachment. It suggests that strengthening the resilience of mothers who have given birth prematurely could enhance maternal bonding.

In previous research, we have identified factors that strengthen post-preterm maternal resilience, including “understanding preterm birth,” “receiving support from others,” “gaining confidence and growing as a parent,” “interacting with the child and perceiving improvements in their growth and health,” “having a more positive outlook on the child's future,” and “feeling less guilt and remorse” [25]. It is crucial to provide interventions tailored to the mother's condition in the early postpartum period to strengthen resilience. Furthermore, Ikeuchi [26] conducted a qualitative study on the emotional trajectory of mothers who gave birth to extremely low birth weight infants, demonstrating that positive events help overcome negative ones. Healthcare professionals, such as midwives present at the birth, must carefully consider the birth experience, which is frequently viewed negatively by mothers. Furthermore, while mother-infant separation is emotionally difficult for mothers, it is vital to support them in building up positive experiences. This includes warmly welcoming mothers to the NICU and involving them in their infant's care to foster maternal confidence. If mothers who experience preterm birth can acquire and strengthen resilience, their coping skills in postpartum crisis and mother-infant bonding will be improved.

Many mothers in the preterm group were forced to undergo hospitalization during pregnancy and faced psychological and physical adversity. Therefore, support based on gestational age is feasible. If preterm birth is anticipated, support measures such as building trust through prenatal visits by pediatricians or NICU nurses can also contribute to increasing resilience. This study revealed that spousal social support is also a factor influencing the mother's bonding after preterm birth. It is believed that mothers after early childbirth feel more valued when their husbands, who can share their pain, recognize the situation [27]. This indicates that from the early postpartum period, it is important to provide support to enable the husband to be the strongest supporter for the mother, in addition to couples counseling, to improve the ability to overcome the crisis.

In this study, EPDS scores were higher in the preterm group than in the term group, consistent with prior research indicating that preterm mothers suffer from postpartum depression [27]. However, no association was found between postpartum depression and mother-infant bonding in the preterm group. This is likely because, although the mean EPDS score for mothers in the preterm group was higher than the term group in this study, it was still below the cutoff value considered to be a risk factor for postpartum depression in the Japanese population. However, previous studies have reported inconsistent results with respect to this association [26,28]. Therefore, psychological support for postpartum depression after preterm birth is required, irrespective of the mother-infant bond, and further investigation is required.

A limitation of this study is that, despite recruiting participants from multiple facilities over approximately two years, the number of preterm infants was small, with only 40 mothers included in the analysis. A larger sample size is needed for more accurate statistical analysis. This study did not include sociodemographic factors in the data or analyses, which may limit our ability to account for confounding factors or chance effects. Furthermore, multiple regression analysis for term infants failed to construct a valid model. It is possible that the factors underlying attachment formation differ between mothers of preterm and full-term infants. Further investigation into these factors and potential confounding variables is necessary. Moving forward, it will be necessary to conduct long-term observational studies that account for differences based on the gestational age of preterm infants and the status of maternal complications. Future efforts should aim to elucidate factors promoting mother-infant bonding and develop maternal support strategies.

## Conclusions

No significant difference in bonding was observed between the preterm and term groups approximately one week after birth. The preterm group had significantly higher scores on acquired resilience. EPDS was significantly higher in the preterm group compared to the term group. Postpartum depression after preterm delivery requires psychological support regardless of the presence or absence of maternal bonding.

This study found that during the first week postpartum, acquired resilience had the greatest impact on the bond formation among mothers after preterm birth, followed by social support from the husband. It suggests that enhancing the resilience of mothers who experienced preterm birth could strengthen maternal bonding. It is critically important to provide interventions tailored to the mother's condition in the early postpartum period and to strengthen her resilience. Spousal support is also a factor influencing the mother's bonding after preterm birth. This indicates that from the early postpartum period, it is important to provide support to enable the husband to be the strongest supporter for the mother.
